# 3D Bioprinting of Novel κ-Carrageenan Bioinks: An Algae-Derived Polysaccharide

**DOI:** 10.3390/bioengineering9030109

**Published:** 2022-03-06

**Authors:** Diana M. C. Marques, João C. Silva, Ana Paula Serro, Joaquim M. S. Cabral, Paola Sanjuan-Alberte, Frederico C. Ferreira

**Affiliations:** 1 Department of Bioengineering and Institute for Bioengineering and Biosciences, Instituto Superior Técnico, Universidade de Lisboa, Av. Rovisco Pais, 1049-001 Lisbon, Portugal; diana.c.marques@tecnico.ulisboa.pt (D.M.C.M.); joao.f.da.silva@tecnico.ulisboa.pt (J.C.S.); joaquim.cabral@tecnico.ulisboa.pt (J.M.S.C.); 2 Associate Laboratory i4HB—Institute for Health and Bioeconomy, Instituto Superior Técnico, Universidade de Lisboa, Av. Rovisco Pais, 1049-001 Lisbon, Portugal; 3 Centre for Rapid and Sustainable Product Development, Polytechnic of Leiria, 2430-038 Marinha Grande, Portugal; 4 Centre of Structural Chemistry, Department of Chemical Engineering, Instituto Superior Técnico, Universidade de Lisboa, Av. Rovisco Pais, 1049-001 Lisbon, Portugal; anapaula.serro@tecnico.ulisboa.pt; 5 Egas Moniz Interdisciplinary Research Centre, Instituto Universitario, Egas Moniz, Quinta da Granja, Monte de Caparica, 2829-511 Caparica, Portugal; 6 Regenerative Medicine and Cellular Therapies, School of Pharmacy, University of Nottingham, University Park, Nottingham NG7 2RD, UK

**Keywords:** bioprinting, marine biomaterials, natural bioinks, algae-based hydrogels, κ-carrageenan

## Abstract

Novel green materials not sourced from animals and with low environmental impact are becoming increasingly appealing for biomedical and cellular agriculture applications. Marine biomaterials are a rich source of structurally diverse compounds with various biological activities. Kappa-carrageenan (κ-c) is a potential candidate for tissue engineering applications due to its gelation properties, mechanical strength, and similar structural composition of glycosaminoglycans (GAGs), possessing several advantages when compared to other algae-based materials typically used in bioprinting such as alginate. For those reasons, this material was selected as the main polysaccharide component of the bioinks developed herein. In this work, pristine κ-carrageenan bioinks were successfully formulated for the first time and used to fabricate 3D scaffolds by bioprinting. Ink formulation and printing parameters were optimized, allowing for the manufacturing of complex 3D structures. Mechanical compression tests and dry weight determination revealed young’s modulus between 24.26 and 99.90 kPa and water contents above 97%. Biocompatibility assays, using a mouse fibroblast cell line, showed high cell viability and attachment. The bioprinted cells were spread throughout the scaffolds with cells exhibiting a typical fibroblast-like morphology similar to controls. The 3D bio-/printed structures remained stable under cell culture conditions for up to 11 days, preserving high cell viability values. Overall, we established a strategy to manufacture 3D bio-/printed scaffolds through the formulation of novel bioinks with potential applications in tissue engineering and cellular agriculture.

## 1. Introduction

The increasing demand for petroleum-based raw materials has been threatening the sustainability of global economy while contributing for climate change. Therefore, natural polymers derived from biological or renewable resources are strongly required to substitute petroleum-based materials and avoid their environmental impact. In tissue engineering (TE), synthetic biocompatible polymers as poly(ethylene glycol and poly-(caprolactone) are widely used [1]. On the other hand, poly(lactic acid) is a bio-based polymer applied as an alternative to petroleum-derived polymers [2]. In fact, recent progresses have been conquered in 3D printing of such materials, as well as other bioderived polyesters such as polyhydroxyalkanoates [3,4]. However, the use of bio-based polymers is not adequate for some TE applications, as cellular agriculture. Thus, there is a need to discover novel environmentally friendly and plant-based materials obtained from naturally available resources to construct a more sustainable future for TE.

Marine biomaterials are rich sources of structurally diverse compounds with various biological activities [5,6]. However, most of them are underutilized [7]. For example, in 2017, Roberto Nisticò explored shellfish biowaste such as chitin and its derived by-products (e.g., chitosan) to develop biomaterials [8]. Moreover, few seaweeds, which are a potential environmentally friendly source of natural components due to their large biomass production without requiring agricultural land, fertilizers, or freshwater; have been exploited for TE applications [9,10,11].

Kappa (κ)-carrageenan (κ-c) is a polysaccharide that can be easily harvested from macroalgae, which can be cultivated at a rate of spread of 250 m/year, providing large quantities of material [12]. Those macroalgae can be found and cultivated on several coastal locations, including the West European coast [13]. This material also presents interesting properties for TE applications due to its gelation properties, mechanical strength, and resemblance to natural glycosaminoglycans (GAGs) [14]. At high temperatures (>50 °C), κ-c exists in solution as a random coil. The cooling of this material leads to helix formation and aggregation of the helical dimers. At lower temperatures, intermolecular electrostatic interactions between the negatively charged sulphate groups of κ-c chains and cations are established, forming a stable 3D network [15]. Moreover, cations can suppress electrostatic repulsion between the negatively charged chains of carrageenan, inducing packing within the aggregate structure, therefore, playing a vital role in the gelation of the κ-c.

The advantage of κ-c over other algae-based biomaterials commonly used in TE, such as alginate, is that κ-c-based materials have been reported to form more robust gels and do not require the presence of a supporting sacrificial solution during 3D printing [16,17], simplifying the bio-/printing process. κ-c can also be easily shaped into different structures using molding techniques. In previous works, hydrogels were casted into different shapes compatible with human mesenchymal stem/stromal cells (hMSCs) and human-induced pluripotent stem cells (hiPSCs) using wax molds [18,19]. Furthermore, alginate gelates in the presence of calcium ions, which is a signaling cation and can induce biological responses depending on the cell type used [20]. Additional gelating agents of alginate hydrogels includes complexing agents and acids [21].

Other promising biomaterials for the 3D manufacturing of scaffolds include decellularized extracellular matrix (dECM). This is a material from animal origin thus, containing valuable biochemical cues such as collagen, fibronectin, laminin, growth factors and GAGs from the native ECM [22,23]. Examples of dECM-based bioprinted scaffolds can be seen in [23], and there are several reviews dedicated to this topic [24,25]. However, such bioinks present several limitations including the requirement for complex decellularization methods, variability amongst individuals [26] and limited printability and mechanical properties [27]. This material might also present several ethical concerns, contrary to algae-based biomaterials. Furthermore, the continuous advances in the cellular agriculture field are motivating the search for sustainable and edible materials obtained from non-animal sources [28]. For this field, algae-based materials represent ideal candidates as they present an optimal equilibrium in terms of sustainability, nutritional value and consumer acceptance [29].

3D bioprinting has emerged as an approach for tissue and organ engineering that could advance organ transplantation and clinical translation of in vitro-engineered constructs [30,31,32]. 3D extrusion bioprinting is the most commonly used bioprinting technique due to the wide range of compatible materials and its ease of operation. 3D extrusion bioprinting consists of the precise deposition of cell-laden bioinks to construct complex and functional tissues, promoting cell ingrowth and viability [33]. Bioprinted scaffolds have the advantage of providing tissue and patient-specific architectures with suitable resolution at the micron scale and controlled deposition of different cell types at very high cell densities [33,34]. In this technique, pneumatic, mechanical, or solenoid systems are applied to a cartridge containing the bioinks (i.e., inks containing cells), allowing to continuously deposit filaments [35,36]. Detailed description of the key bioink properties and bioprinting parameters can be found elsewhere [37]. κ-c has been previously explored in 3D bioprinting. However, it is often blended with other materials. Indeed, to date no bioinks based on pristine κ-c have been developed. A double network hydrogel was 3D printed by combining κ-c and polyacrylamide [38], showing high strength and mechanical properties [39]. The reported approaches to print pristine κ-c hydrogels, based on an in-situ temperature-induced gelation [40] or UV crosslinking [41], required conditions that are not compatible with cell bioprinting due to the impact of high temperatures (>37 °C) on cell viability and exposure to high intensity UV light that could induce cytotoxic events [42]. Bioprinting of κ-c has only been achieved on composites containing other natural materials obtained from animal sources. For instance, one study reports the fabrication of scaffolds of methacrylamide-modified gelatine and methacrylate κ-c compatible with adipose tissue-derived stem cells (ASCs) [41,43]. However, the use of materials from animal origin could present regulatory, cultural, or ethical concerns.

In this work, two strategies to process pristine κ-c are proposed in the manufacturing of complex constructs by 3D printing and 3D bioprinting, respectively. The research methodology follows a two-step approach. In the first step, deionized water is used to develop and characterized a κ-c ink. However, in the second step culture media is used on a bioink formulation to provide the proper salt balance and nutrients to support cell viability. The κ-c concentration was adjusted according the aqueous matrix used. Moreover, initially, κ-c hydrogels were casted on molds and characterized. This was followed by an optimization of the printability conditions and parameters and to validate the cytocompatibility of the material and the bioprinting process, cell studies using L929 mouse fibroblasts were performed. Once this strategy was established, this material was then used in the formulation of bioinks. To the best of our knowledge, this study presents for the first time a strategy for the 3D bioprinting of pristine κ-c bioinks with optimized conditions to support cell growth.

## 2. Materials and Methods

### 2.1. Algae-Based Inks Formulation and Printing

Kappa (κ-)-carrageenan (κ-c) was purchased from Sigma-Aldrich (St. Louis, MI, USA) without further treatment. The κ-c solutions were prepared by dissolving κ-c in ultrapure water (MilliQ, Millipore, Merck, Darmstadt, Germany) under continuous stirring in a hotplate (Stuart, Cole-Palmer, Vernon Hills, IL, USA) at 80 °C until fully dissolved. Different concentrations were prepared including 10 g L^−1^, 15 g L^−1^, 20 g L^−1^, and 25 g L^−1^ κ-c. The inks were denominated with the following nomenclature 10κ-c, 15κ-c, 20κ-c, and 25κ-c, respectively. Once dissolved, solutions were kept at 37 °C prior to printing. For the 3D bioprinting, 6κ-c, 9κ-c, and 12κ-c inks with 6 g L^−1^, 9 g L^−1^, 12 g L^−1^ κ-c, respectively, dissolved in media were also evaluated. The procedure of cell addition to the bioinks is described in Section 2.5.4. Bio-/printing was performed using a custom made microextrusion-based 3D printing system as described previously [44]. Briefly, this system consists of a three-axis dispensing robot Fisnar F4200N.2, (FISNAR, Germantown, WI, USA) and a pneumatic dispensing unit (DC100 High Precision Dispenser, Ellsworth, Glasgow, UK) interfaced with a personal computer. The syringe barrels, syringe barrel adaptor, end caps, pistons, and dispensing tips were also provided by Ellsworth. The digital models were uploaded to the robot using the Fisnar RobotEdit software (FISNAR, Germantown, WI, USA). Parameters including printing speed, applied pressure, and distance between layers were modified to find the optimal printing conditions.

### 2.2. Hydrogel Scaffold Characterization

Cylindrical molds with 8 mm diameter and 4–8 mm in height were used as support to cast hydrogels. The height was varied depending on the requirements of the characterization techniques. These molds were 3D printed by fused deposit modelling using a Prusa i3 MK3S 3D extruder (PRUSA Research, Prague, Czech Republic) loaded with a PLA filament. During the casting process, the molds were filled with the desired solution volume at room temperature until gelation was completed. After this, molds were carefully removed using tweezers. The incubation time was approximately 10 min at RT and the gelation was enhanced by adding a drop of KCl 0.5 M.

#### 2.2.1. Swelling Degree

Casted 10κ-c, 15κ-c, 20κ-c, and 25κ-c cylindrical hydrogels with 8 mm in diameter and 8 mm in height were stored in phosphate buffer saline (PBS) and incubated at room temperature for seven days. Measurements of swollen hydrogel weights were recorded every two days. For measurements, PBS excess was carefully removed from hydrogels, and the hydrogels were weighed. Swelling (%) was calculated from Equation (1):

(1) $$\mathrm{Swelling}\;\left(\%\right)=\frac{\left(W_{S}-W_{0}\right)}{W_{0}}\times 100$$

where W_0_ is the initial weight of the hydrogel and W_s_ is the weight of the swollen hydrogel. All measurements were performed in triplicate (*n* = 3).

#### 2.2.2. Water Content

Casted hydrogels were prepared following the previous procedure. Water content of the hydrogels was estimated by weighting the samples to obtain the initial mass. Materials were then dried overnight at 43.5 °C under vacuum. The water content (%) was estimated according to Equation (2):

(2) $$\mathrm{Water}\;\mathrm{Content}\;\left(\%\right)=\frac{\left(W_{0}-W_{d}\right)\;}{W_{0}}\times 100$$

where W_0_ is the initial weight of the hydrogel and W_d_ is the weight of the dried hydrogel.

#### 2.2.3. Compression Mechanical Tests

A uniaxial compression test was performed using a Univert (CellScale Biomaterials Testing, Waterloo, ON, Canada) load frame equipped with a 10 N load cell. Hydrogels casted following the previous methods were used for this assay, specimens were cylindrical (6–8 mm in diameter and 6–8 mm in height, *n* = 5), and the crosshead speed was set constant displacement rate at 3 mm min^−1^ during the test. Stress-strain curves were obtained considering Equations (3) and (4). The linear region of stress-strain curves was selected to calculate the Young’s modulus (Equation (5)), which corresponds to the stress-strain curve’s 0–15% linear strain region.

(3) $$\mathrm{Stress}=\;\sigma =\frac{F}{A}$$

where F (Newton, N) is applied force, and A (mm^2^) is cross-section area.

(4) $$\mathrm{Strain}=\varepsilon =\frac{\Delta L}{L_{o}}$$

where ΔL (mm) is displacement and Lo (mm) is the initial height.

(5) $$\mathrm{Youn}g\prime \;s\;\mathrm{Modulus}=\frac{\sigma }{\varepsilon }$$

where σ (N mm^−2^) is stress and ε (non-dimensional) is strain. Since 1 N mm^−2^ = 10^6^ Pascal (Pa) = 1000 kPa conversions were performed to discuss the Young’s Modulus in kPa units.

### 2.3. Rheological Characterization and Temperature Dependance of Bio-/Inks

The rheological properties of the inks were determined using an MCR 92 modular compact rheometer (Anton Paar, Graz, Austria). The rheological assessment was conducted using a cone-plate geometry with a cone diameter of 50 mm, and a constant measurement gap of 0.1 mm, resulting in a sample volume of 0.5 mL. The instrument was calibrated to analyze the viscoelastic region.

To estimate the sol-gel transition temperature of the bio-/inks, temperature sweeps were performed. The viscosity was determined at a shear rate of 50 s^−1^ with temperatures varying from 37 to 20 °C (cooling). The assay ran over 300 s with a rate of 0.2 °C s^−1^. The temperature range was selected assuming the value of 37 °C for the printing conditions and RT for the post-printing conditions. Each measurement was performed in triplicate (*n* = 3).

The elastic and viscous behavior of the materials were characterized by recording the storage modulus (G′), and the loss modulus (G″) as a function of time under an oscillating time sweep test with a frequency of 1 Hz at 25 °C, for ten minutes. The time dependence of G′ and G″ was evaluated in the 15κ-c ink. The gelation time corresponds to the time point where G″ intersects G′. Each measurement was performed in triplicate (*n* = 3).

### 2.4. Semi-Quantification of Printability

The inks printability was estimated based on the concept of circularity of an enclosed area (A) within a given perimeter (L), which is defined by Equation (6):

(6) $$C=\frac{4\pi A}{L^{2}}$$

Printability of the inks to achieve square-shaped pores was quantified from microscopy images taken with a Leica DMI3000B (Leica Microsystems, Wetzlar, Germany). The printability factor (Pr) was estimated as described elsewhere [45], based on the ratio of the theoretical (C′) to the experimental (C) circularity. For a square shape, C′ is equal to π/4. Therefore, Pr was measured considering the pore perimeter (L) and the pore area (A) using Equation (7):

(7) $$\mathrm{Pr}=\frac{\pi }{4}.\frac{1}{C}=\frac{L^{2}}{16A}$$

A perfect crosslinked ink that forms ideal square-shaped pores corresponds to Pr = 1, where Pr < 1 corresponds to under-gelated inks and rounded pore corners, and Pr > 1 to over-gelated inks.

The following inks were evaluated for printability: 10κ-c, 15κ-c, 20κ-c, 25κ-c, 9κ-c ink, and 9κ-c bioink. The 3D printed scaffolds exhibited a squared-mesh structure with the side dimensions of 10 × 10 mm^2^ and 1–3 layers printed in height, with 0.05 mm distance between layers. A 20-gauge (inner diameter of 0.5 mm) or a 23-gauge (inner diameter of 0.6 mm) QuantX™ blunt end dispense tips (FISNAR, Germantown, WI, USA) were used at a printing speed of 25 mm s^−1^. The Pr values were determined by analyzing the optical microscopy images of printed constructs using ImageJ software (ImageJ 1.51f, National Institutes of Health, Bethesda, MD, USA) to determine the perimeter and area of interconnected channels (*n* = 5).

### 2.5. Cell Culture, Bioprinting and Biocompatibility Assessments 

#### 2.5.1. Cell Culture and Passaging

L929 mouse fibroblasts were purchased from Sigma and cultured in Dulbecco’s Modified Eagle’s Medium (DMEM, ThermoFischer, Waltham, MA, USA) with 10% Fetal Bovine Serum (FBS, Gibco, ThermoFischer, Waltham, MA, USA) and 1% antibiotics (penicillin/streptomycin, Sigma Aldrich) solution. Cells were incubated at 37 °C in a 5% CO_2_ atmosphere using a T-25 flask (Falcon BD, Franklin Lakes, NJ, USA). The culture medium was changed every two days and passaged when 80% confluency was reached. The passaging was accomplished using a trypsin/EDTA solution (Sigma-Aldrich). Cells were counted on a hemocytometer using the Trypan Blue (ThermoFischer, Waltham, MA, USA) exclusion method to determine cell viability and total number of cells.

#### 2.5.2. Cell Seeding onto 3D Printed Scaffolds

For cell seeding experiments, hydrogels were printed as scaffolds with side dimensions of 10 × 10 mm, and three layers. A 20-gauge blunt end dispense tip (Fisnar) and a printing speed of 25 mm s^−1^ were used. After printing the scaffolds, sol-gel transition was performed by immersing the scaffolds in a potassium chloride (KCl) 0.5 M bath. The printed scaffolds were then placed in a 12-well plate and were sterilized with 1% (*v*/*v*) antibiotic-antimycotic (solution with 10,000 units/mL of penicillin, 10,000 µg/mL of streptomycin, and 25 µg/mL of Amphotericin B; Sigma Aldrich) in PBS overnight. Cells were seeded on the scaffold by adding a 15 µL drop containing 50,000 cells on each scaffold. Structures were incubated for 1 h inside the laminar flow cabinet after seeding to promote initial cell attachment before carefully adding culture medium until covering the whole scaffold. Subsequently, cell-seeded scaffolds were incubated at 37 °C in a 5% CO₂ atmosphere. The medium was supplemented with filter-sterilized 100 µL KCl 0.5 M and changed every 2–3 days for 8 days.

#### 2.5.3. Cell Proliferation Assay

Cellular proliferation was assessed using an Alamar Blue™ Cell Viability Reagent (Thermo Fisher Scientific, Waltham, MA, USA) on days 2 and 7 after seeding on the previous materials to get an indirect estimate of cell numbers in the 3D printed scaffolds. Alamar Blue™ reagent (100 µL) was incubated for 2 h on the different materials at 37 °C and 5% CO₂. After this, the fluorescence intensity was measured in the aforementioned plate reader at an excitation/emission wavelength of 560/590 nm. Hydrogels with no cells were used as blank controls. Samples were measured in triplicate. 

#### 2.5.4. Incorporation of Cells into the Bioink

The bioprinting process was performed using the aforementioned instrument. In this case, cells were suspended at a concentration of 3,400,000 cells mL^−1^. 500 μL of this cell suspension was incorporated into the 9κ-c bioinks by mixing thoroughly to reach a final concentration of 500,000 cells per mL of bioink. This cell concentration was selected based on previous works [46]. Ring structures with a radius of 4 mm and three layers were bioprinted. The 3D bioprinting process took place inside a laminar flow cabinet, and bioprinting parameters consisted of a distance between layers of 0.05 mm, a printing pressure ranging 8–10 psi, and a printing speed set at 25 mm s^−1^. A 23-gauge blunt end dispense tip was used. The scaffolds were bioprinted in culture plates (Falcon) and incubated in 5 mL of culture medium, supplemented with 100 µL of KCl 0.5 M. The medium was changed every 2–3 days for a period of 11 days.

#### 2.5.5. Cell Viability Assay

A Live/Dead assay was performed to establish the viability of seeded and bioprinted fibroblasts. For this, cells were washed in PBS and incubated for 30 min in 1 µM acetoxymethyl (AM) calcein solution (Sigma Aldrich #C1359) in PBS to stain viable cells (green), while dead cells were stained with 5 µM ethidium homodimer I (Sigma Aldrich #E1903) in PBS (red). Fluorescence images were taken on a Leica DMI3000B fluorescence microscope (Leica Microsystems). Three representative images were obtained for each structure (N = 3, *n* = 3). The resulting images were used to quantify live and dead cells using ImageJ software (ImageJ 1.51f, National Institutes of Health, Bethesda, MD, USA).

#### 2.5.6. Staining of Cellular Components 

Fluorescence staining of L929 fibroblasts was performed by fixing cells in 4% paraformaldehyde (PFA, Sigma) in PBS for 30 min at room temperature and washed in PBS following permeabilization with 0.01% Triton-X 100 (Sigma) in PBS for 8 min. Nucleus staining was performed using 5 µg mL^−1^ Hoechst 33342 (Thermo Fisher Scientific) in PBS for 10 min at 37 °C. Alexa Fluor 488^®^ Phalloidin (Thermo Fisher Scientific) at a dilution factor of 1:150 in PBS was applied for 30 min at room temperature. A final washing step in PBS was performed prior to imaging in a fluorescence microscope. Cell clusters were quantified via ImageJ software and classified as follows: very small (10–20 cells), small (20–40 cells), medium (40–60 cells), large (60–80 cells), and very large (80–100 cells).

#### 2.5.7. Structure Stability of 9κ-c Bioink

3D printed squared meshes constructed with the 9κ-c bioink were incubated at different temperatures (20, 37, 40, and 50 °C) and supplemented with 100 μL of KCl 0.5M to evaluate structure stability. The selection of this bioink was based on the results obtained from the rheological characterization (See Section 3.4). The dimensions of the squared-mesh structures corresponded to 20 × 20 mm^2^ and 1–3 layers, with a 0.05 mm distance between layers. A 20-gauge blunt end dispense tips (Fisnar) were used at a printing speed of 20 mm s^−1^.

### 2.6. Statistical Analysis

Data is presented as mean values ± standard deviations. Each experiment was conducted in triplicate (*n* = 3) unless stated otherwise. Statistical significance was performed through t-student tests using GraphPad Prism 9 (GraphPad, San Diego, CA, USA).

## 3. Results and Discussion

### 3.1. Properties of Casted κ-Carrageenan (κ-c) Hydrogels

**Figure 1:**
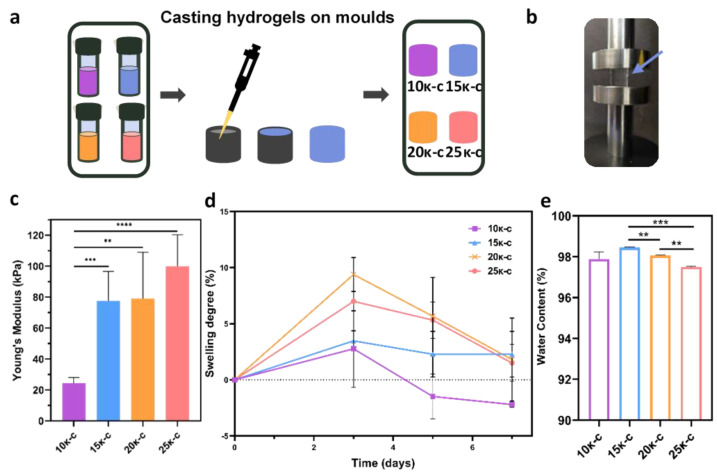
Properties of casted 10κ-c, 15κ-c, 20κ-c, and 25κ-c hydrogels. Process of (**a**) casting hydrogels on molds using previously formulated κ-based inks. (**b**) Casted hydrogels were placed on the mechanical test apparatus for a compression test. Arrow indicates the location of the sample. (**c**) Young’s modulus determined for the different hydrogels. (**d**) Percentage of swelling degree on all samples on day zero, three, five, and seven after preparation. (**e**) Water content determination on all hydrogels. Data are shown as mean ± SD (*n* = 5). Statistical significance was assessed using t-student analysis, showing non-significant *p*-values (*p*-value ≥ 0.05) and values with different significances (*p* ** < 0.01; *p* *** < 0.001; *p* **** < 0.0001).

Initially, 10κ-c, 15κ-c, 20κ-c, and 25κ-c hydrogels were casted into as described in Figure 1a with 6–8 mm in diameter and 6–8 mm in height to assess their mechanical properties, swelling degree and water content. 

The assessment of the mechanical properties of the gelated hydrogels was performed by a uniaxial compression test (Figure 1b). The Young’s modulus values of each sample were estimated from the linear region of stress-strain curves (Figure S1). As expected, the hydrogel with the lowest κ-c concentration presented a significantly lower Young’s modulus value, corresponding to 24.26 kPa. Higher κ-c concentrations led to higher Young’s modulus values corresponding to 77.65, 79.08, and 99.90 kPa for 15κ-c, 20κ-c, and 25κ-c, respectively. This trend can be seen in Figure 1c. However, values were not statistically different in the case of 15κ-c, 20κ-c, and 25κ-c. These values are similar to those previously reported in the literature [47]. The robustness of the constructs was also observed through mechanical manipulation of the structures (using tweezers and a spatula), with no permanent deformations noted (Figure S2). This characteristic is quite relevant for tissue engineering applications, as samples might require certain manipulation.

Once the mechanical characterization was completed, we proceeded to evaluate the swelling degree and water content of the different samples. Hydrogels started swelling immediately after immersion in PBS and reached equilibrium three days after immersion in all samples. This corresponded to 2.77, 3.47, 9.39, and 7.00 swelling percentage for the 10κ-c, 15κ-c, 20κ-c, and 25κ-c, respectively (Figure 1d). The correlation between swelling and κ-c concentration has also been observed in other studies, where higher κ-c concentrations led to an increased swelling [48]. We hypothesize that this initial swelling is due to an ionic potential difference. After that, internal and external ions reach an equilibrium state, stabilizing the swelling rate [49]. This behavior was only observed in the 15κ-c hydrogels. The other materials presented a slight decrease in scaffold weight, revealing water loss, possible due to degradation of the hydrogel or damage associated with sample manipulation. 

The water content of the scaffolds (Figure 1e) showed a slight but statistically significant reduction of the water content with increasing κ-c concentrations. Higher κ-c concentrations are able to promote more helix formation and aggregation of the material, increasing the density of the scaffold and 3D network. All scaffolds showed a water content above 97%. These results are in line with previous works where water content of κ-c hydrogels was reported as 99% [47]. It is important to note that there has been reported a strong correlation between materials hydration and cell adhesion [50]. 

### 3.2. Characterization of the Printability of κ-c Inks

A preliminary assessment of the printing conditions and parameters was performed, and we determined that it was not possible to print 3D structures with the 10κ-c, 20κ-c, and 25κ-c inks. The 10κ-c ink resulted in a low-viscosity formulation where printed structures lacked any self-support, due to its higher water content. In the case of the 20κ-c ink, gelation was taking place inside the printing cartridge, leading to the extrusion of a partially gelated material that resulted in deformed structures. This effect was more evident in the case of the 25κ-c ink, where this material was not extrudable. The results of this preliminary assessment can be seen in Figure S3. Therefore, the 15κ-c was selected for the following printing experiments and its rheological behavior was assumed as ideal when compared to the previous inks and the subsequent bioink formulations (See Section 3.4), (Figure S4).

**Figure 2:**
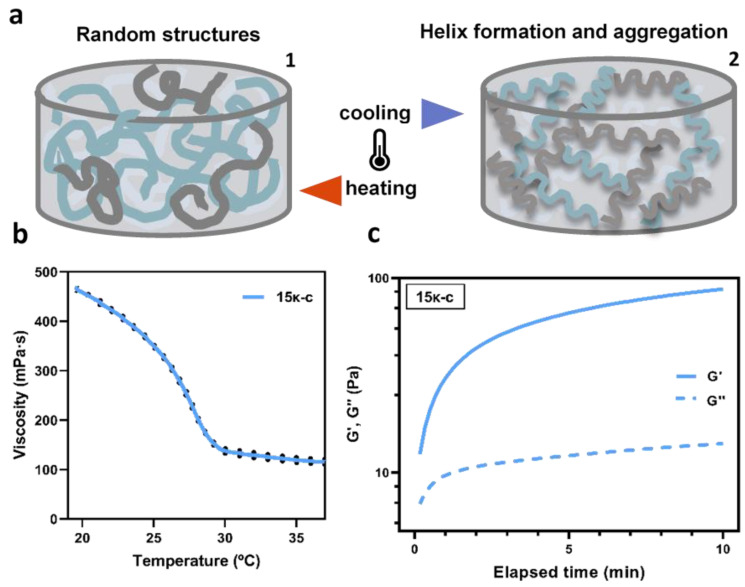
(**a**) Schematic representation of the reversible and temperature-dependent gelation process of κ-c-based inks. (1) Aqueous solutions of κ-c existing as random structures at high temperatures. Light grey, dark grey, and blue represents the same components in different planes. (2) Cooling process leads to helix formation and aggregation, forming a stable 3D network. (**b**) Gelation kinetics of the 15κ-c ink showing the storage (G′) and loss moduli (G″) over time at room temperature. Solid lines indicate the average values, and the doted lines indicate the SD. (**c**) Assessment of the viscosity as a function of temperature. Three samples were analyzed in each assay (*n* = 3).

Since the gelation process of the inks is highly dependent on the temperature, we proceeded to investigate this further to optimize the bio-/printing conditions. At high temperatures, κ-c exists in the solution as a random coil. However, a cooling process can lead to helix formation and aggregation, forming a stable 3D network (Figure 2a). The rheological behavior of the 15κ-c ink was evaluated as a function of temperature from 37 °C to 20 °C. This range of temperatures was selected to simulate the conditions of the bio-/printing and post bio-/printing processes. Results are shown in Figure 2b. The curves indicate that the sol-gel transition (coil-to-helix) is taking place when temperature reaches 29 °C. Above 29 °C, the viscosity of the solutions remained stable in the range of 110.24–158.62 mPa due to the higher presence of disorganized random coils. Below 29 °C, the viscosity abruptly increased, reaching values of 468.74 mPa suggesting the formation and organization of the κ-c helix. This behavior was already reported in the literature for similar studies on the κ-c rheological properties [51,52]. Other authors also explained that sol-gel transition temperatures increase with increasing polymer concentration. This transition is also highly dependent on the presence of potassium, calcium, and cesium ions [53,54]. 

The viscoelastic behavior of the 15κ-c ink was also characterized by estimating the storage modulus (G′) and the loss modulus (G″) as a function of time for 10 min, at 25 °C (Figure 2c). G′ represents the elastic portion of the viscoelastic behavior and reflects the elastic performance of material when deformed. The G″ refers to the viscous portion of the viscoelastic behavior, which reflects the flow of a material while it is deformed. When a 3D network is not fully developed, there are many non-interacting segments such as random coil and loops within the hydrogel. These are mobile domains and less effective elastically. The G″ is attributable to these non-interacting segments. During gelation, the establishment of more network interactions results in G′ values higher than G″, where solid-like behavior dominates the viscoelastic properties of the hydrogel [55]. The point where G′ = G″ indicates the gelation point. In Figure 2c we can observe that for the results obtained, G′ > G″ at all time points, and thus no intersection point is observed, indicating that helix formation had already occurred to a certain degree prior to measurements. This behaviour is typical of gel-like products [56]. This experiment confirms the high temperature-dependency of the materials and the need to carefully control this parameter during printing. 

**Figure 3:**
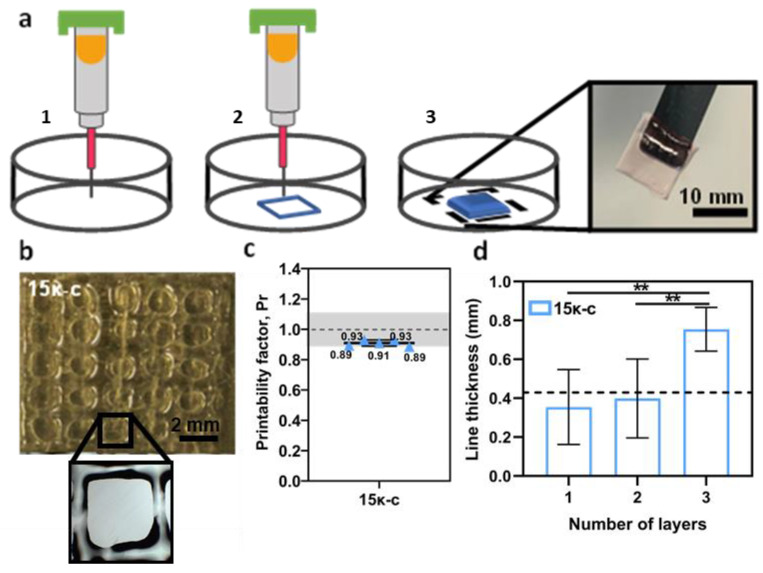
(**a**) Schematic representation of the extrusion printing process of κ-c based hydrogels. (1) A syringe printing directly to a petri dish. (2) Extrusion printing of κ-c based inks. (3) This method can be used to 3D print a 10 × 10 mm scaffold. Scale bar 10 mm. (**b**) Optical microscopy images of a 3D printed 15κ-c square mesh and optical microscopy image of one of the pores used to determine the printability of the material. (**c**) Printability factor of the 15κ-c ink with individual printability factor values. The grey region marks the range of adequate ink printability (from 0.9 to 1.1) as described in [57]. Data is shown as mean ± SD (*N* = 3, *n* = 5). (**d**) Determination of the line thickness over three different layers of the 15κ-c printed hydrogel. The expected line thickness is marked with a black dotted line (0.5 mm = needle inner diameter). Data are shown as mean ± SD (*N* = 3, *n* = 5). Statistical significance was assessed using a t-student analysis (*p* ** < 0.01).

Once the temperature window was established, further optimization of the printing parameters was performed. For this, different printing speeds and pressures were assessed. In our case, the optimized values corresponded to 25 mm s^−1^ and 2–4 psi for printing speed and pressure, respectively. A layer distance of 0.05 mm was kept between printed layers. Using these parameters, we were able to fabricate 10 × 10 mm scaffolds as seen in Figure 3a. A printability assessment was performed to measure the fidelity of the printing process. Printability, namely the ability of the inks to fabricate 3D structures with exemplary integrity, is a representative criterion to evaluate the physical properties of the inks. Printability quantification was determined by calculating the printability factor (Pr) from the pores of printed meshes (Figure 3b). This calculation determined that the printability of the 15κ-c inks fell within the acceptable region (0.9–1.1 Pr) (Figure 3c). We also evaluated how the line thickness of the constructs was affected by the printing of additional layers. There are no significant variations in line thickness from printing 1 or 2 layers. However, after printing three layers the line thickness increases from 0.354 mm to 0.754 mm (Figure 3d). Overall, these results indicate that the 15κ-c is adequate for 3D printing. 

### 3.3. Preliminary Assessment of Cell Viability, Proliferation and Morphology 

**Figure 4:**
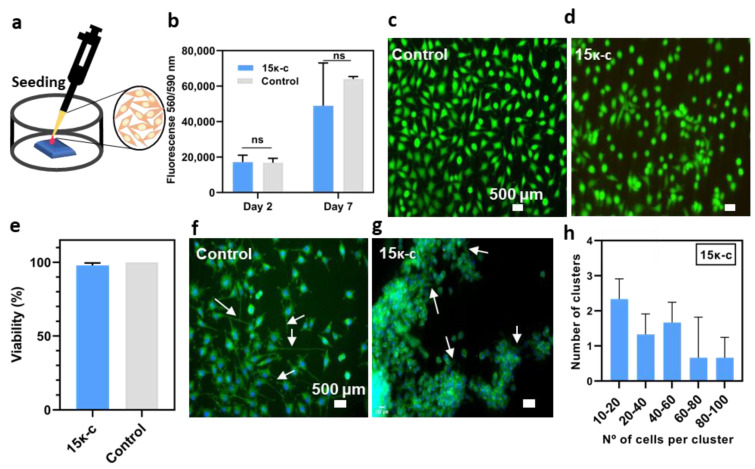
Characterization of cells seeded on the 3D printed hydrogels. (**a**) Schematic representation of the cell seeding process on the 10 × 10 mm printed hydrogels. (**b**) Assessment of cell proliferation using an AlamarBlue™ test on day two and day seven after seeding. Each column shows the mean ± SD of three independent experiments (*n* = 3). Statistical significance was assessed using a two-way ANOVA analysis. Fluorescence microscopy images of fibroblasts seeded on (**c**) control substrates and (**d**) κ-c scaffolds after Live/Dead staining with ethidium homodimer 1 (dead cells) and calcein-AM (viable cells). (**e**) Percentage of viable cells calculated from fluorescence microscopy images (*n* = 3). Statistical significance was assessed using t-student analysis, (ns *p* ≥ 0.05). Hoechst/Phalloidin staining to the cells cultured on (**f**) control substrates and (**g**) 15κ-c printed scaffolds. Arrow indicates cell adhesion sites to the substrate. (**h**) Quantification of the number of cells per cluster presented in the representative fluorescence images. Data are shown as mean ± SD (*n* = 3).

In order to preliminary evaluate the capacity of cells to grow and proliferate on our printed structures, L929 mouse fibroblasts were seeded onto 15κ-c 3D printed scaffolds (10 mm × 10 mm) and cultured for eight days as described in Figure 4a. An AlamarBlue™ assay was performed on day two and day seven to evaluate cell proliferation, since this assay allows a direct correlation between cell number and cell metabolic activity. When compared to controls (cells seeded on regular culture plates), cells seeded on the 3D printed scaffolds presented slightly lower average values of fluorescence on day seven. However, statistically, both conditions exhibit similar values (Figure 4b).

In order to confirm that there were no cytotoxic effects associated to the material and/or supplementation with KCl 0.5 M in the culture media, a viability assay was performed using live/dead fluorescence probes on day eight. The results of this on the printed scaffolds and control surface can be seen in Figure 4c,d. It is important to note that in the case of the printed scaffolds there seems to be more cells exhibiting a round morphology. This could be due to the lack of adhesion sites in the κ-c hydrogels. Regardless of the morphology, most of the cells were stained with the green probe indicating that they were viable. The quantification of the viability was performed from the previous images, where viabilities were quite comparable between both samples, being 97.84% and 100% for κ-c and control surfaces, respectively (Figure 4e). 

Further visualization of the cellular components was performed following a Hoechst-Phalloidin staining to observe the nuclei and actin filaments of the cytoskeleton of the cells. The presence of adhesion focal points at the cell periphery was observed on both conditions. However, this was more evident in the control substrate (Figure 4f,g). When compared to controls, cells seeded on 3D printed scaffolds showed inferior cytoskeleton organization and defects in cell spreading. Moreover, these cells tend to form cell clusters favoring cell-to-cell interactions, and to have a rounded cell morphology, which we hypothesize that is due to the lack of adhesion points in the material. This causes fibroblasts to undergo an adaptation period on the materials, possibly until they are able to produce their own ECM [58]. These clusters were quantified and sorted by size as seen in Figure 4h. The criteria to classify these clusters were previously described in Section 2.5.6. Zhang et al. showed that the addition of κ-c to hydrophobic substrates improves cell attachment and proliferation of seeded ATDC5 cells [59]. However, we observed that our scaffolds promote cell adhesion in a clustered manner. These clusters led to higher cell density on the 3D printed scaffolds, associated with a round shape due to lower spreading area and higher circularity. Nevertheless, from these studies we conclude that cells can grow and remain viable when cultured on κ-c based hydrogels.

### 3.4. Bioprinting of 3D Structures Using Pristine κ-c Bionks

The previous results based on the 15κ-c ink demonstrated optimal printability with this material and its ability to support cell growth, providing a strategy to easily process κ-c structures by 3D printing that could be applied to several bioengineering applications. Bioprinting with this material has not been achieved previously and therefore, in order to advance this further, the previous formulation was reassessed to increase its compatibility with the bioprinting process.

Despite displaying optimal printability, the 15κ-c ink uses ultrapure water as solvent, in the absence of the proper ionic, nutrients and signaling factor compositions, thus compromising cell viability during the bioink formulation. In order to solve this, bioinks were formulated in media. κ-c concentration had also to be optimized since the presence of ions in the media could trigger the gelation of the materials prior to bioprinting. For this reason, lower κ-c concentrations were evaluated in the bioink formulations: 6κ-c, 9κ-c, and 12κ-c.

**Figure 5:**
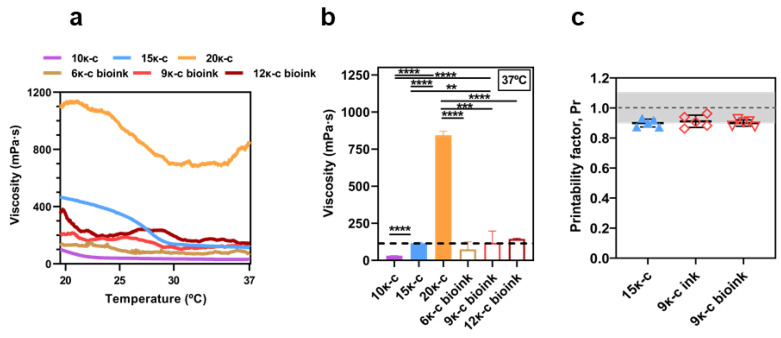
(**a**) Assessment of the mean value of viscosity as a function of temperature of 10κ-c, 15κ-c, 20κ-c, 6κ-c bioink, 9κ-c bioink, and 12κ-c bioink and their (**b**) specific viscosity at 37 °C. Three samples were analyzed in each assay (*n* = 3). Statistical significance was assessed using a t-student analysis (*p* ** < 0.01; *p* *** < 0.001; *p* **** < 0.0001). (**c**) Printability factor of the 15κ-c ink compared with the 9κ-c with (bioink) and without cells (ink). The grey region marks the range of adequate ink printability (from 0.9 to 1.1) [57]. Data are shown as mean ± SD (*n* = 3, *n* = 5).

The temperature dependence of these formulations was compared to the previous inks. As it can be seen in Figure 5a, the graphs from the bioink formulations present a more irregular trend. We hypothesize that this might be due to the presence of gelated and non-gelated areas in the bioinks triggered by the presence of salts in the media in contrast to the uniform gelation taking place at the inks prepared in ultrapure water, despite lower concentrations are used. 

Nevertheless, at the bio-/printing temperature (37 °C), both the 15κ-c ink and the 9κ-c bioink presented similar viscosity values, corresponding to 118.87 mPa s and 130.83 mPa s, respectively (Figure 5b). At room temperature, the rheological behavior of such inks differs, showing that 9κ-c bioink is suitable for bioprinting but loses stability at room temperature printing. These results were confirmed by mechanical compression tests, as shown in Figure S5. This is not the case of the other formulations assessed, and we can therefore conclude that the 9κ-c bioink presents the most similar behavior to our previously optimized 15κ-c ink during the bio-/printing process. At lower temperatures, viscosity values differ (Figure S4) in these two formulations. However, this does not affect the printability or stability of the bio-/printed structures.

Evaluation of the printability was carried out on the 9κ-c bioink prior to and after the addition of cells following the previous procedures (Figure S6). The printability results indicated two main findings: (1) addition of cells to the bioink composition does not affect printability of the material (Figure 5c), and (2) printability values are comparable to the 15κ-c ink. 

**Figure 6:**
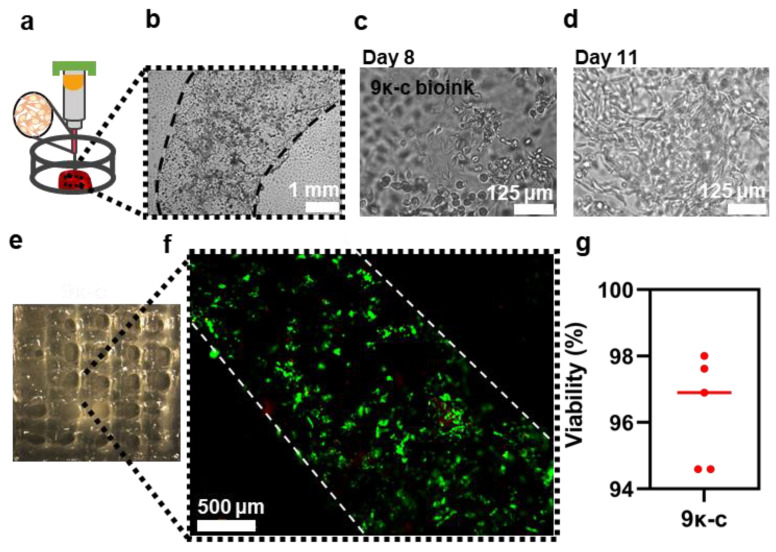
Cell characterization on the 3D bioprinted constructs. (**a**) Schematic representation of the bioprinting process of rings with a radius of 4 mm. Brightfield image of bioprinted fibroblasts in 9κ-c bioink with dotted black line indicating the edge of the structures on (**b**) day zero, (**c**) day eight, and (**d**) day eleven. (**e**) Optical microscopy images of a 3D bioprinted 9κ-c square mesh and respective (**f**) fluorescence microscopy images of the fibroblasts encapsulated on such bioprinted structures (**f**) after Live/Dead staining with ethidium homodimer 1 (dead cells) and calcein-AM (viable cells). Percentage of viable cells (**f**) within the bioprinted scaffolds calculated from fluorescence images of bioprinted fibroblasts after the Live/Dead staining using the 9κ-c bioink (**f**,**g**).

Considering the previous results, 3D bioprinted rings and meshes were fabricated using the 9κ-c bioink containing fibroblasts as described in Figure 6a. These cells remained stable in culture for up to 11 days. In Figure 6b we can observe the structures after bioprinting by optical microscopy. Dotted lines indicate the borders of the bioprinted ring. As cells were suspended in the bioinks they presented a round morphology. In order to preserve the stability of the bioprinted structures at 37 °C, we have evaluated the capacity of our structures to maintain their shape at different temperatures in the presence and absence of KCl supplementation. As it can be seen in Figure S7, 9κ-c structures were compromised in both conditions after culture above 40 °C. At lower temperatures, including 37 °C, only those structures previously supplemented with KCl maintained their shape and morphology. It is therefore essential to add KCl in the culture media in order to preserve the 3D bioprinted structures in culture conditions.

After 8 days in culture, some of the encapsulated cells started to spread inside the material, while other preserved a round morphology (Figure 6c). Interestingly, after 11 days in culture, the encapsulated cells populated most of the hydrogel surface and presented a more elongated morphology than cells seeded on printed scaffolds (Figure 6d). This phenomenon has also been reported in the literature, as bioprinting enhances cell distribution within the material.

Previous studies have also suggested that fibroblasts encapsulated in κ-c based bioinks have the inherent potential to self-organize into 3D aggregated spheroids [60]. This behavior was also observed after live/dead staining. Fluorescence microscopy images show that most of the cells are viable and that these cells can reorganize into relatively complex structures after bioprinting (Figure 6e,f). More images are available in Figure S8. Bioprinted cells showed a high viability estimated from the previous images (Figure 6g). The presence of dead cells was negligible, which implies that the hydrogels are cytocompatible and non-toxic. The high cell viability of 97% falls on the range of the values reported by other studies on extrusion-based bioprinting [61]. Cell viabilities above 90% were previously reported for κ-c gelatine hydrogel constructs [43]. Importantly, this demonstrates that the proposed bioinks developed in this work have the potential to be used for the development of complex 3D tissues, supporting cellular growth while maintaining high printability.

## 4. Conclusions

In this work, we propose for the first time a novel formulation of pure κ-c-based bio-/inks that allows the bio-/printing of complex 3D structures while maintaining high cell viability and reorganization. Initially hydrogel properties were assessed, including Young’s modulus, swelling ratio, and water content. These properties indicated that κ-c was a suitable material for tissue engineering applications. Rheological characterization of the materials was performed to understand the effect of temperature on the gelation of the material and kinetics. Different 3D structures were developed following bio-/printing methods, presenting robust mechanical properties allowing for the structures self-support. This considerably simplifies bioprinting methods as it is not required the use of sacrificial supporting materials, often used in most of the current bioprinting strategies. L929 mouse fibroblasts were incorporated into the bioinks formulation, and they remained viable during and after the bioprinting process. Furthermore, the morphology and organization of these cells was superior when they were encapsulated in the bioprinted structures than when seeded on printed scaffolds. Overall, this study presents a novel strategy to produce 3D complex tissues in vitro based on algae-based materials with low environmental impact.

## Data Availability

All data from this study are available from the authors upon request.

## Supplementary Materials

The following supporting information can be downloaded at: https://www.mdpi.com/article/10.3390/bioengineering9030109/s1. Figure S1: Stress-strain curves for the four κ-based inks; Figure S2: 3D printed structures using the 15κ-c ink; Figure S3: 3D printed squared meshes of (**a**) 10κ-c and (**b**) 20κ-c; Figure S4: Specific viscosity of 10κ-c, 15κ-c, 20κ-c inks and 6κ-c, 9κ-c, and 12κ-c bioinks at 25 °C and at 20 °C. Three samples were analysed in each assay (*n* = 3); Figure S5. (**A**) Stress-strain curves of 9κ-c bioink during a compression test, at room temperature, and respective (**B**) Young modulus value comparing o the 15κ-c ink; Figure S6: 3D bio-/printed squared meshes used in the calculation of the printability factor of 9κ-c ink and the 9κ-c bioink and higher magnification images of the pores used for this; Figure S7: Structure stability of 3D printed 9κ-c bio-inks at different temperatures (20, 37, 40, and 50 °C), with (blue circle) or without the supplementation of KCl (black circle). Different levels were de-fined to describe the stability of each scaffold (3—very stable; 2—stable; 1—not stable); Figure S8: Fluorescence microscopy images of the fibroblasts encapsulated on bioprinted structures after Live/Dead staining with ethidium homodimer 1 (dead cells) and calcein-AM (viable cells).
