# Supplementary material for: 3D Bioprinting of Novel κ-Carrageenan Bioinks: An Algae-Derived Polysaccharide
Source: Bioengineering (Basel). 2022 Mar 6;9(3):109. doi: 10.3390/bioengineering9030109 (PMC8945127; doi:10.3390/bioengineering9030109)
Supplement: Supplementary file 1 [file bioengineering-09-00109-s001.zip › bioengineering-1545507-supplementary.pdf]

Article

3D Bioprinting of Novel κ-Carrageenan Bioinks: An Algae-Derived Polysaccharide

Diana M. C. Marques [1,2], João C. Silva [1,2,3], Ana Paula Serro [4,5], Joaquim M. S. Cabral [1,2], Paola Sanjuan-Alberte [1,2,6,*] and Frederico C. Ferreira [1,2,*]

1 Department of Bioengineering and Institute for Bioengineering and Biosciences, Instituto Superior Técnico, Universidade de Lisboa, Av. Rovisco Pais, 1049-001 Lisbon, Portugal; diana.c.marques@tecnico.ulisboa.pt (D.M.C.M.); joao.f.da.silva@tecnico.ulisboa.pt (J.C.S.); joaquim.cabral@tecnico.ulisboa.pt (J.M.S.C.)
2 Associate Laboratory i4HB—Institute for Health and Bioeconomy, Instituto Superior Técnico, Universidade de Lisboa, Av. Rovisco Pais, 1049-001 Lisbon, Portugal
3 Centre for Rapid and Sustainable Product Development, Polytechnic of Leiria, 2430-038 Marinha Grande, Portugal
4 Centre of Structural Chemistry, Department of Chemical Engineering, Instituto Superior Técnico, Universidade de Lisboa, Av. Rovisco Pais, 1049-001 Lisbon, Portugal; anapaula.serro@tecnico.ulisboa.pt
5 Egas Moniz Interdisciplinary Research Centre, Instituto Universitario, Egas Moniz, Quinta da Granja, Monte de Caparica, 2829-511 Caparica, Portugal
6 Regenerative Medicine and Cellular Therapies, School of Pharmacy, University of Nottingham, University Park, Nottingham NG7 2RD, UK
* Correspondence: paola.sanjuanalberte2@nottingham.ac.uk (P.S.-A.); frederico.ferreira@tecnico.ulisboa.pt (F.C.F.)

Citation: Marques, D.M.C.; Silva, J.C.; Serro, A.P.; Cabral, J.M.S.; Sanjuan-Alberte, P.; Ferreira, F.C. 3D Bioprinting of Novel κ-Carrageenan Bioinks: An Algae-Derived Polysaccharide. *Bioengineering* **2022**, *9*, 109. https://doi.org/10.3390/bioengineering9030109

Academic Editors: Chaozong Liu, Saman Naghieh, Gabriella Lindberg and Gary Chinga Carrasco

Received: 23 December 2021
Accepted: 02 March 2022
Published: 06 March 2022

Publisher's Note: MDPI stays neutral with regard to jurisdictional claims in published maps and institutional affiliations.

Copyright: © 2022 by the authors. Submitted for possible open access publication under the terms and conditions of the Creative Commons Attribution (CC BY) license (https://creativecommons.org/licenses/by/4.0/).

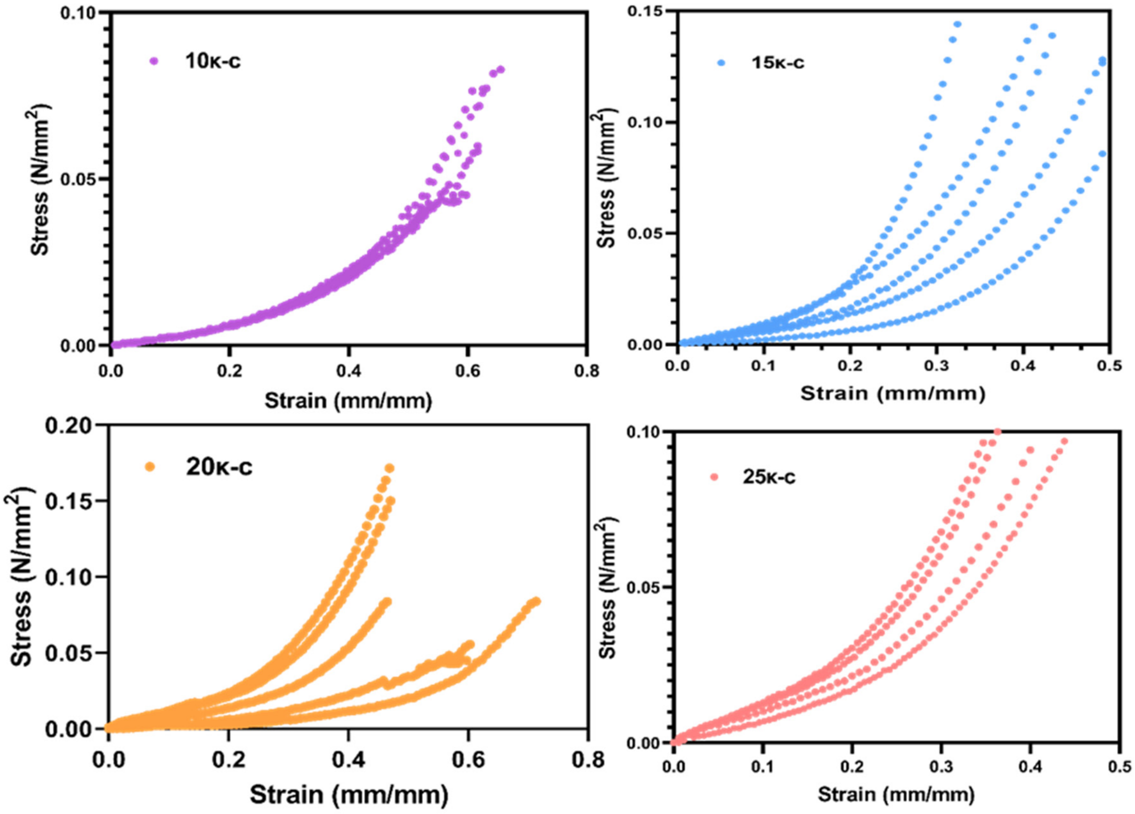

Figure S1. Stress-strain curves for the four κ-based inks.

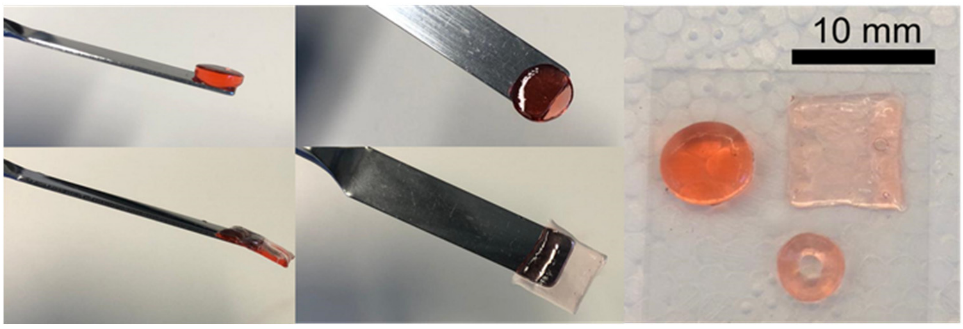

Figure S2. 3D printed structures using the 15κ-c ink.

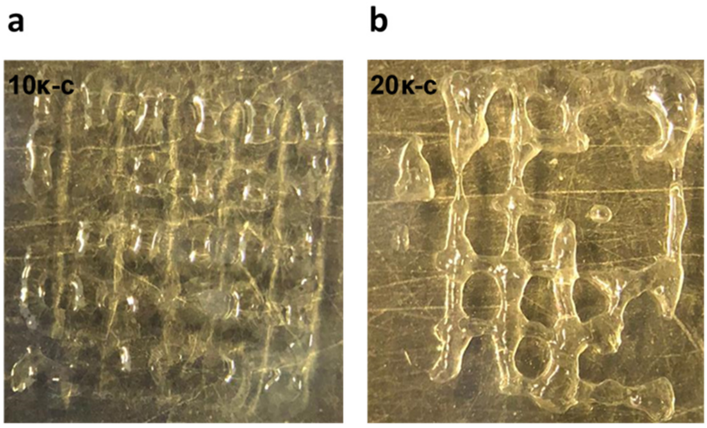

Figure S3. 3D printed squared meshes of **(a)** 10κ-c and **(b)** 20κ-c.

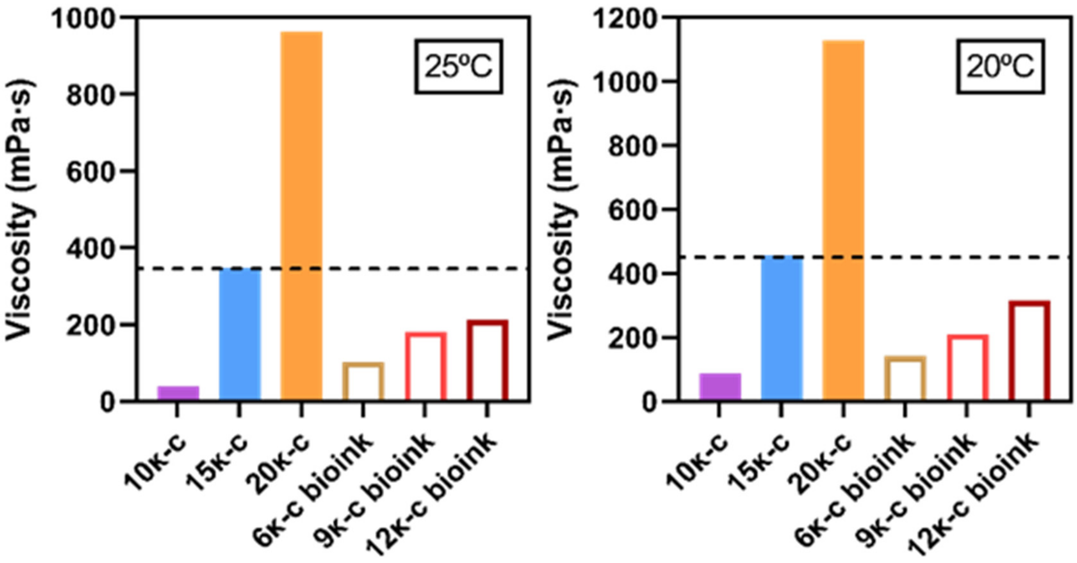

Figure S4. Specific viscosity of 10κ-c, 15κ-c, 20κ-c inks and 6κ-c, 9κ-c, and 12κ-c bioinks at 25ºC and 20ºC. Three samples were analysed in each assay (*n*=3).

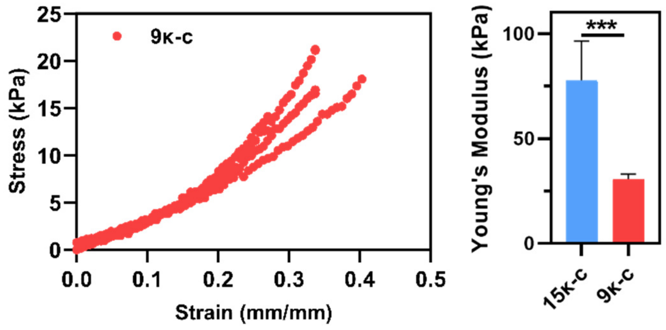

Figure S5. A) Stress-strain curves of 9κ-c bioink during a compression test, at room temperature, and respective B) Young modulus value comparing o the 15κ-c ink.

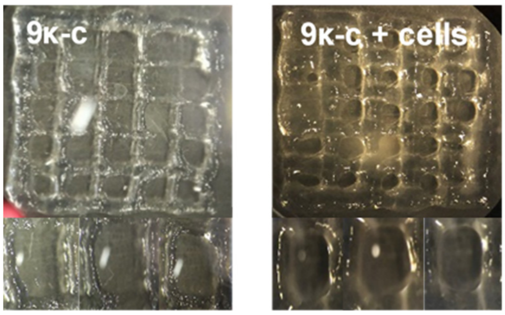

Figure S6. 3D bio-/printed squared meshes used in the calculation of the printability factor of 9κ-c ink and the 9κ-c bioink and higher magnification images of the pores used for this.

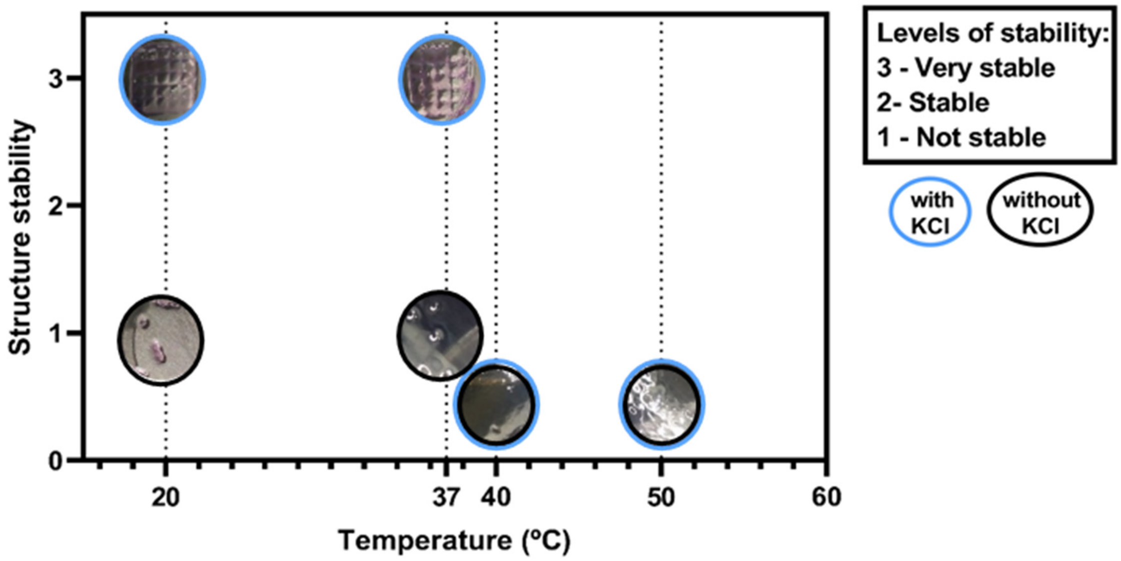

Figure S7. Structure stability of 3D printed 9κ-c bio-inks at different temperatures (20, 37, 40, and 50 ºC), with (blue circle) or without the supplementation of KCl (black circle). Different levels were defined to describe the stability of each scaffold (3 – very stable; 2 – stable; 1 – not stable).

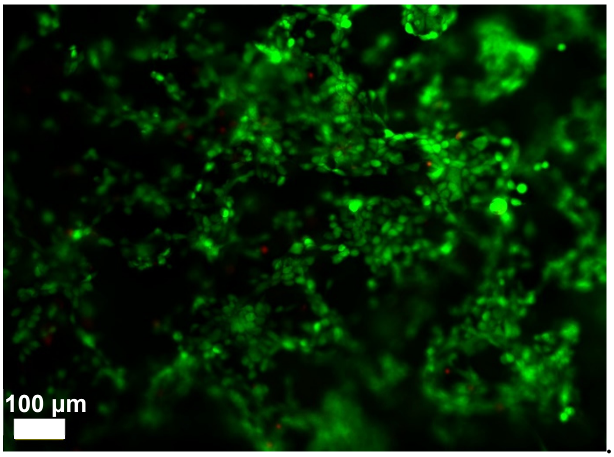

Figure S8. Fluorescence microscopy images of the fibroblasts encapsulated on bioprinted structures after Live/Dead staining with ethidium homodimer 1 (dead cells) and calcein-AM (viable cells).
